# Insights into the Role of *Erysipelotrichaceae* in the Human Host

**DOI:** 10.3389/fcimb.2015.00084

**Published:** 2015-11-20

**Authors:** Nadeem O. Kaakoush

**Affiliations:** School of Biotechnology and Biomolecular Sciences, Faculty of Science, University of New South WalesSydney, NSW, Australia

**Keywords:** *Erysipelotrichaceae*, gastrointestinal, microbiota, metabolic disorders, inflammatory bowel diseases, colorectal cancer

Understanding the human gut microbiota has garnered interest from researchers and clinicians to pharmaceutical companies looking at novel mechanisms to manipulate the microbiota for the benefit of the host. Studies on the gut microbiota can be loosely characterized into three areas that include investigating the microbiota's role in the physiology of the healthy gut, in the establishment of gastrointestinal disease, and in extra-intestinal manifestations. With deep sequencing technologies now in routine use in the research environment, novel members of the gut microbiota are coming to light, and our understanding of this complex ecosystem and its relationship to the host is slowly improving.

## Importance of *Erysipelotrichaceae* in humans

Reports documenting a potential role for the bacterial family *Erysipelotrichaceae*, which belongs to the Firmicutes phylum, in host physiology and/or disease are on the rise. However, more often than not, these organisms are mentioned in passing, despite the fact that members of this bacterial family appear to be highly immunogenic and can potentially flourish post-treatment with broad spectrum antibiotics (Zhao et al., 2013; Palm et al., 2014; Dinh et al., 2015). For example, Palm and colleagues who developed a method to sort and sequence members of the intestinal microbiota based on coating with immunoglobulin A (IgA), termed IgA-SEQ, found one member of the *Erysipelotrichaceae* to be highly coated by IgA relative to other members of the gut microbiota (Palm et al., 2014). More recently, Ding and colleagues observed that the relative abundance of Erysipelotrichi positively correlated with tumor necrosis factor alpha (TNF) levels in a study investigating patients who had chronic HIV infection and were receiving suppressive antiretroviral therapy and HIV-uninfected controls (Dinh et al., 2015). Further, Zhao and colleagues found an increase in the levels of *Erysipelotrichaceae* incertae sedis in mice treated with high doses of gentamicin (Zhao et al., 2013).

## *Erysipelotrichaceae* and inflammation-related gastrointestinal diseases

The importance of *Erysipelotrichaceae* in inflammation-related disorders of the gastrointestinal tract is highlighted by the fact that they have been found to be enriched in colorectal cancer. For example, their abundance levels were found to be increased in the lumen of colorectal cancer patients as compared to healthy controls (Chen et al., 2012), and to be significantly higher in the tumor group of an animal model of 1, 2-dimethylhydrazine-induced colon cancer (Zhu et al., 2014).

Changes in the levels of *Erysipelotrichaceae* in patients with inflammatory bowel diseases (IBD) or animal models of IBD have also been observed; however, the evidence does not appear to be consistent (Craven et al., 2012; Dey et al., 2013; Gevers et al., 2014; Labbé et al., 2014; Palm et al., 2014; Schaubeck et al., 2015). Craven and colleagues identified ileitis-associated shifts toward *Erysipelotrichaceae* in C57BL6 mice infected with *Toxoplasma gondii* or *Giardia muris* (Craven et al., 2012), while Schaubeck and colleagues observed significant increases in the abundance of *Erysipelotrichaceae* in mice that develop a TNF-driven Crohn's disease (CD)-like transmural inflammation (Schaubeck et al., 2015). These findings are of interest, given the association between *Erysipelotrichaceae* and TNF levels (Dinh et al., 2015).

In contrast, Dey and colleagues found that patients who experienced recurrence of CD had significantly lower levels of *Erysipelotrichaceae* (Dey et al., 2013), and similarly, Gevers and colleagues found a decreased abundance of Erysipelotrichales in patients within new-onset CD (Gevers et al., 2014). These findings are supported by a study by Labbe and colleagues who analyzed publicly available metagenomic datasets of IBD patients and controls for bile metabolizing genes, and found that the abundance of bile salt hydrolase genes originating from Firmicutes taxa including *Erysipelotrichaceae* was significantly reduced in IBD as compared to healthy controls (Labbé et al., 2014).

These results would suggest inter-host variation, with inflammatory mouse models having higher levels of *Erysipelotrichaceae*, and patients with IBD having lower levels. Two possible explanations for this include the inherent differences in the gut microbiota of mice and humans and/or differences in their innate immune responses upon sensing bacterial ligands (Zschaler et al., 2014; Nguyen et al., 2015). However, Palm and colleagues did not observe a significant difference in the abundance levels of a specific immunogenic *Erysipelotrichaceae* species between IBD patients and controls (Palm et al., 2014). Nonetheless, when the authors infected germ-free mice with a consortium of bacteria that were classified as highly coated by IgA (containing *Erysipelotrichaceae* spp.), these mice developed a more severe colitis upon treatment with dextran sodium sulfate than mice colonized by an IgA-negative consortium of bacteria (Palm et al., 2014). Thus, the inconsistencies in the results related to IBD could potentially be attributed to a lack of proper characterization of taxa belonging to the *Erysipelotrichaceae* family. For example, species within *Erysipelotrichaceae* may have diverse immunogenicity profiles or respond differently to inflammation within the gut.

## *Erysipelotrichaceae* and metabolic disorders

Perhaps the strongest evidence for a role for *Erysipelotrichaceae* in human disease comes from studies related to metabolic disorders. Early work on this topic showed a bloom of species belonging to *Erysipelotrichaceae* (classified as Mollicutes at the time of that study) in diet-induced obese animals (Turnbaugh et al., 2008). In addition, higher levels of *Erysipelotrichaceae* in obese individuals (Zhang et al., 2009), and a drop of approximately 2.5-fold in the abundance of *Erysipelotrichaceae* taxa in a hamster model of hypercholesterolemia treated with an extract to improve cholesterol homeostasis (Martínez et al., 2009) have been observed. Subsequent studies have confirmed the association between this bacterial family and lipidemic profiles within the host. For example, Zhang and colleagues identified four different lineages within *Erysipelotrichaceae* to respond differently to diet or host health phenotypes (Zhang et al., 2010), while Fleissner and colleagues observed an increase of *Erysipelotrichaceae* in mice on high-fat or western diet (Fleissner et al., 2010). Further, Spencer and colleagues showed that the abundance of Erysipelotrichi were positively associated with changes in liver fat in female subjects who were placed on diets in which choline levels were manipulated (Spencer et al., 2011).

More recently, in an important study examining the microbiota of hamsters whose cholesterol metabolism was modulated using dietary plant sterol esters, Martinez and colleagues provided strong evidence for a correlation between the levels of *Erysipelotrichaceae* and host cholesterol metabolites (Martínez et al., 2013). In addition to this, Etxeberria and colleagues observed that supplementation of the flavonol quercetin inhibited the growth of *Erysipelotrichaceae* (Etxeberria et al., 2015), which is particularly interesting given that flavonoids have been suggested to assist in weight loss (Hurt and Wilson, 2012).

## Nutrition studies provide further insights into the role of *Erysipelotrichaceae* in the gut

Nutrition studies further support the influence of dietary fat on the abundance of *Erysipelotrichaceae*. Harris and colleagues identified an accumulation of a specific taxon within *Erysipelotrichaceae* in mice with liver injury associated with parenteral nutrition, and observed a marked reduction in liver injury also corresponding to a decrease in the abundance of the same *Erysipelotrichaceae* taxon following antibiotic treatment (Harris et al., 2014). The authors determined that the fluctuations in abundance of *Erysipelotrichaceae* corresponded to the soy oil based-lipid emulsion from the parenteral nutrition solution (Harris et al., 2014).

**Figure 1:**
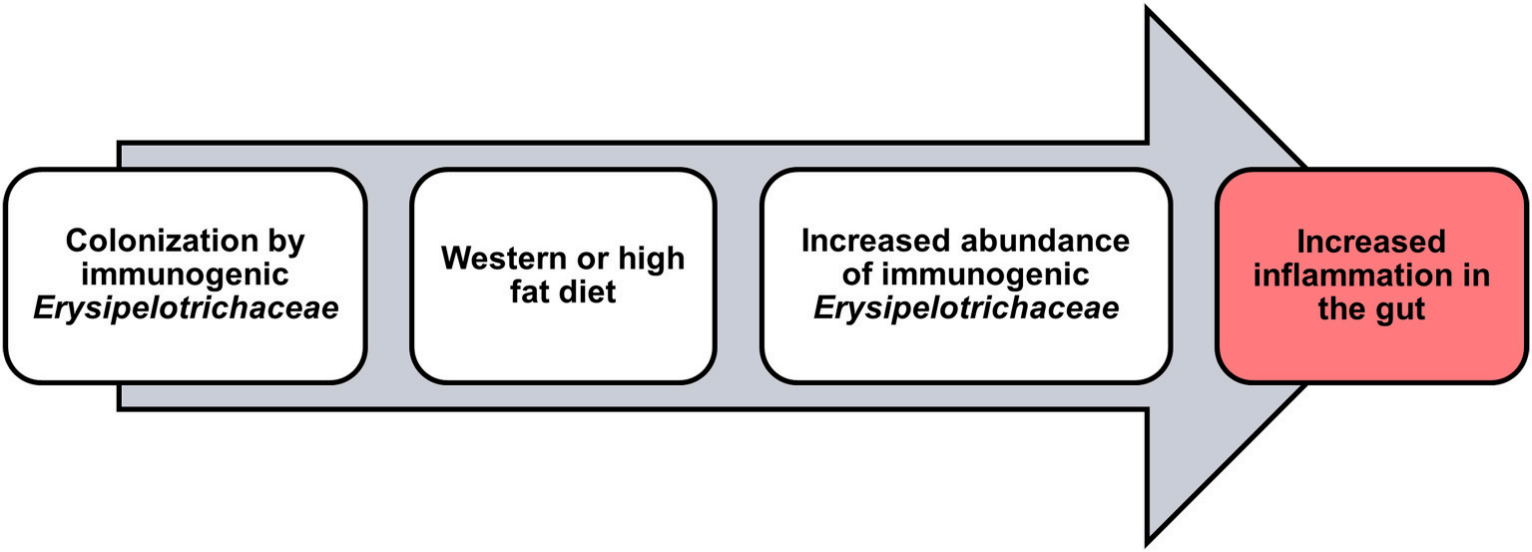
**Potential role for ***Erysipelotrichaceae*** in inflammation within the host**.

In support of these findings, our group has recently observed a decrease in the abundance of *Erysipelotrichaceae* in CD patients undergoing low-fat (~13.5 g/1000 Kcal; Nahidi et al., 2013) exclusive enteral nutrition (EEN) therapy (Kaakoush et al., 2015). This is of interest when taken together with the findings of Li and colleagues who showed that EEN therapy was associated with a significant decrease in visceral fat area in patients with CD (Li et al., 2014). Indeed, Gassull and colleagues have concluded that the type of dietary fat may be important for the efficacy of enteral nutrition in CD (Gassull et al., 2002). Given this, it is no surprise that IBD patients often exhibit differences in their levels of cholesterol as compared to healthy subjects (Agouridis et al., 2011), and infliximab maintenance therapy targeting TNF in CD patients, leads to an increase in cholesterol levels and a significant increase in abdominal fat tissue (Parmentier-Decrucq et al., 2009). With all the above evidence in mind, the fluctuations in *Erysipelotrichaceae* levels in IBD patients are likely to be associated with patient lipid or cholesterol phenotypes and/or patient diet, but may still have a significant impact on disease presentation and/or activity (Figure 1).

## Conclusions

Taken together, there is strong evidence supporting an association between *Erysipelotrichaceae* and host lipid metabolism, warranting additional investigation into the metabolic profiles of these organisms, and the influence they have on the host. Partial loss of fatty acid biosynthesis has been reported in the genomes of some members of *Erysipelotrichaceae* (e.g., *Erysipelothrix rhusiopathiae*; Kwok et al., 2014); thus, it would be of interest to determine if these metabolic characteristics have any impact on their role within the host. Further, the finding that specific taxa within *Erysipelotrichaceae* may be correlated to inflammation (Dinh et al., 2015), while others are highly immunogenic (Palm et al., 2014) adds weight to the importance of properly characterizing this bacterial family, and may eventually provide promising microbial targets to combat metabolic disorders. It is worth noting, however, that current evidence associating this bacterial family with disease is correlative, and studies examining the direct impact that changes in the abundance of *Erysipelotrichaceae* have on the host are required.

## Author contributions

NK conceived the idea and wrote the manuscript.

### Conflict of interest statement

The author declares that the research was conducted in the absence of any commercial or financial relationships that could be construed as a potential conflict of interest.

## References

[B1] Agouridis A. P. Elisaf M. Milionis H. J. (2011). An overview of lipid abnormalities in patients with inflammatory bowel disease. Ann. Gastroenterol. 24, 181–187. 24713706 PMC3959314

[B2] Chen W. Liu F. Ling Z. Tong X. Xiang C. (2012). Human intestinal lumen and mucosa-associated microbiota in patients with colorectal cancer. PLoS ONE 7:e39743. 10.1371/journal.pone.0039743 22761885 PMC3386193

[B3] Craven M. Egan C. E. Dowd S. E. McDonough S. P. Dogan B. Denkers E. Y.. (2012). Inflammation drives dysbiosis and bacterial invasion in murine models of ileal Crohn's disease. PLoS ONE 7:e41594. 10.1371/journal.pone.0041594 22848538 PMC3404971

[B4] Dey N. Soergel D. A. Repo S. Brenner S. E. (2013). Association of gut microbiota with post-operative clinical course in Crohn's disease. BMC Gastroenterol. 13:131. 10.1186/1471-230X-13-131 23964800 PMC3848607

[B5] Dinh D. M. Volpe G. E. Duffalo C. Bhalchandra S. Tai A. K. Kane A. V.. (2015). Intestinal microbiota, microbial translocation, and systemic inflammation in chronic HIV infection. J. Infect. Dis. 211, 19–27. 10.1093/infdis/jiu409 25057045 PMC4326316

[B6] Etxeberria U. Arias N. Boqué N. Macarulla M. T. Portillo M. P. Martínez J. A.. (2015). Reshaping faecal gut microbiota composition by the intake of trans-resveratrol and quercetin in high-fat sucrose diet-fed rats. J. Nutr. Biochem. 26, 651–660. 10.1016/j.jnutbio.2015.01.002 25762527

[B7] Fleissner C. K. Huebel N. Abd El-Bary M. M. Loh G. Klaus S. Blaut M. (2010). Absence of intestinal microbiota does not protect mice from diet-induced obesity. Br. J. Nutr. 104, 919–929. 10.1017/S0007114510001303 20441670

[B8] Gassull M. A. Fernández-Bañares F. Cabré E. Papo M. Giaffer M. H. Sánchez-Lombraña J. L.. (2002). Fat composition may be a clue to explain the primary therapeutic effect of enteral nutrition in Crohn's disease: results of a double blind randomised multicentre European trial. Gut 51, 164–168. 12117873 10.1136/gut.51.2.164 PMC1773299

[B9] Gevers D. Kugathasan S. Denson L. A. Vázquez-Baeza Y. Van Treuren W. Ren B.. (2014). The treatment-naive microbiome in new-onset Crohn's disease. Cell Host Microbe 15, 382–392. 10.1016/j.chom.2014.02.005 24629344 PMC4059512

[B10] Harris J. K. El Kasmi K. C. Anderson A. L. Devereaux M. W. Fillon S. A. Robertson C. E.. (2014). Specific microbiome changes in a mouse model of parenteral nutrition associated liver injury and intestinal inflammation. PLoS ONE 9:e110396. 10.1371/journal.pone.0110396 25329595 PMC4203793

[B11] Hurt R. T. Wilson T. (2012). Geriatric obesity: evaluating the evidence for the use of flavonoids to promote weight loss. J. Nutr. Gerontol. Geriatr. 31, 269–289. 10.1080/21551197.2012.698222 22888842

[B12] Kaakoush N. O. Day A. S. Leach S. T. Lemberg D. A. Nielsen S. Mitchell H. M. (2015). Effect of exclusive enteral nutrition on the microbiota of children with newly diagnosed Crohn's disease. Clin. Transl. Gastroenterol. 6, e71. 10.1038/ctg.2014.21 25588524 PMC4418409

[B13] Kwok A. H. Li Y. Jiang J. Jiang P. Leung F. C. (2014). Complete genome assembly and characterization of an outbreak strain of the causative agent of swine erysipelas–*Erysipelothrix rhusiopathiae* SY1027. BMC Microbiol. 14:176. 10.1186/1471-2180-14-176 24993343 PMC4105556

[B14] Labbé A. Ganopolsky J. G. Martoni C. J. Prakash S. Jones M. L. (2014). Bacterial bile metabolising gene abundance in Crohn's, ulcerative colitis and type 2 diabetes metagenomes. PLoS ONE 9:e115175. 10.1371/journal.pone.0115175 25517115 PMC4269443

[B15] Li Y. Zhu W. Gong J. Zuo L. Zhang W. Gu L.. (2014). Influence of exclusive enteral nutrition therapy on visceral fat in patients with Crohn's disease. Inflamm. Bowel Dis. 20, 1568–1574. 10.1097/MIB.0000000000000114 24983977

[B16] Martínez I. Perdicaro D. J. Brown A. W. Hammons S. Carden T. J. Carr T. P.. (2013). Diet-induced alterations of host cholesterol metabolism are likely to affect the gut microbiota composition in hamsters. Appl. Environ. Microbiol. 79, 516–524. 10.1128/AEM.03046-12 23124234 PMC3553751

[B17] Martínez I. Wallace G. Zhang C. Legge R. Benson A. K. Carr T. P.. (2009). Diet-induced metabolic improvements in a hamster model of hypercholesterolemia are strongly linked to alterations of the gut microbiota. Appl. Environ. Microbiol. 75, 4175–4184. 10.1128/AEM.00380-09 19411417 PMC2698331

[B18] Nahidi L. Leach S. T. Mitchell H. M. Kaakoush N. O. Lemberg D. A. Munday J. S.. (2013). Inflammatory bowel disease therapies and gut function in a colitis mouse model. Biomed. Res. Int. 2013:909613. 10.1155/2013/909613 24027765 PMC3763566

[B19] Nguyen T. L. Vieira-Silva S. Liston A. Raes J. (2015). How informative is the mouse for human gut microbiota research? Dis. Model Mech. 8, 1–16. 10.1242/dmm.017400 25561744 PMC4283646

[B20] Palm N. W. de Zoete M. R. Cullen T. W. Barry N. A. Stefanowski J. Hao L.. (2014). Immunoglobulin A coating identifies colitogenic bacteria in inflammatory bowel disease. Cell 158, 1000–1010. 10.1016/j.cell.2014.08.006 25171403 PMC4174347

[B21] Parmentier-Decrucq E. Duhamel A. Ernst O. Fermont C. Louvet A. Vernier-Massouille G.. (2009). Effects of infliximab therapy on abdominal fat and metabolic profile in patients with Crohn's disease. Inflamm. Bowel Dis. 15, 1476–1484. 10.1002/ibd.20931 19291781

[B22] Schaubeck M. Clavel T. Calasan J. Lagkouvardos I. Haange S. B. Jehmlich N.. (2015). Dysbiotic gut microbiota causes transmissible Crohn's disease-like ileitis independent of failure in antimicrobial defence. Gut. [Epub ahead of print]. 10.1136/gutjnl-2015-309333 25887379 PMC4752651

[B23] Spencer M. D. Hamp T. J. Reid R. W. Fischer L. M. Zeisel S. H. Fodor A. A. (2011). Association between composition of the human gastrointestinal microbiome and development of fatty liver with choline deficiency. Gastroenterology 140, 976–986. 10.1053/j.gastro.2010.11.049 21129376 PMC3049827

[B24] Turnbaugh P. J. Bäckhed F. Fulton L. Gordon J. I. (2008). Diet-induced obesity is linked to marked but reversible alterations in the mouse distal gut microbiome. Cell Host Microbe 3, 213–223. 10.1016/j.chom.2008.02.015 18407065 PMC3687783

[B25] Zhang C. Zhang M. Wang S. Han R. Cao Y. Hua W.. (2010). Interactions between gut microbiota, host genetics and diet relevant to development of metabolic syndromes in mice. ISME J. 4, 232–241. 10.1038/ismej.2009.112 19865183

[B26] Zhang H. DiBaise J. K. Zuccolo A. Kudrna D. Braidotti M. Yu Y.. (2009). Human gut microbiota in obesity and after gastric bypass. Proc. Natl. Acad. Sci. U.S.A. 106, 2365–2370. 10.1073/pnas.0812600106 19164560 PMC2629490

[B27] Zhao Y. Wu J. Li J. V. Zhou N. Y. Tang H. Wang Y. (2013). Gut microbiota composition modifies fecal metabolic profiles in mice. J. Proteome Res. 12, 2987–2999. 10.1021/pr400263n 23631562

[B28] Zhu Q. Jin Z. Wu W. Gao R. Guo B. Gao Z.. (2014). Analysis of the intestinal lumen microbiota in an animal model of colorectal cancer. PLoS ONE 9:e90849. 10.1371/journal.pone.0090849 24603888 PMC3946251

[B29] Zschaler J. Schlorke D. Arnhold J. (2014). Differences in innate immune response between man and mouse. Crit. Rev. Immunol. 34, 433–454. 10.1615/critrevimmunol.2014011600 25404048

